# Supplementary material for: Dopamine D2 receptor regulates cortical synaptic pruning in rodents
Source: Nat Commun. 2021 Nov 8;12:6444. doi: 10.1038/s41467-021-26769-9 (PMC8576001; doi:10.1038/s41467-021-26769-9)
Supplement: Supplementary file 1 — Supplementary information [file 41467_2021_26769_MOESM1_ESM.pdf]

Supplementary information

Dopamine D2 receptor regulates cortical synaptic pruning in rodents

Ya-Qiang Zhang, Wei-Peng Lin, Li-Ping Huang, Bing Zhao, Cheng-Cheng Zhang and Dong-Min Yin

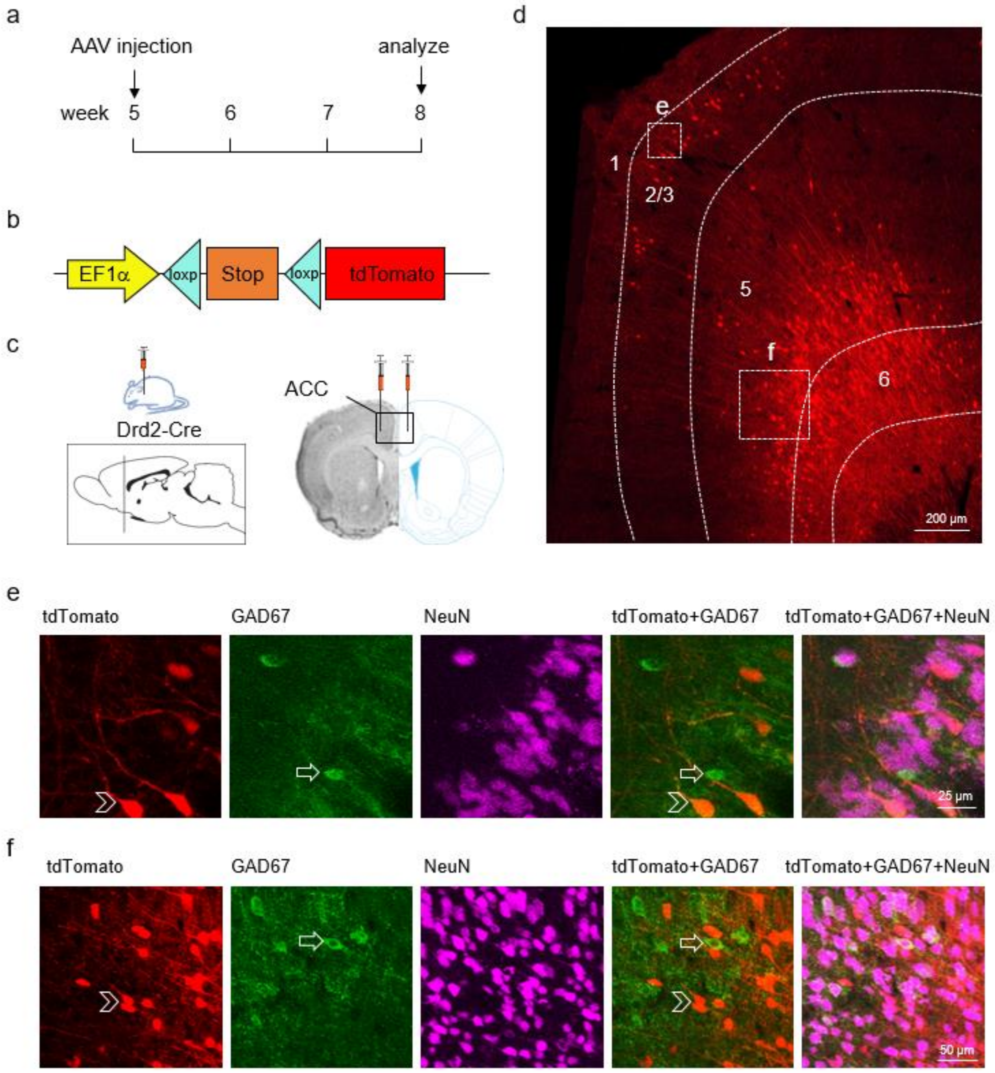

Supplementary Fig. 1 Cellular Expression pattern of Drd2 in rat ACC.
a Experimental design. Three weeks after injection of Cre-dependent tdTomato reporter AAV, the heterozygous Drd2-Cre rats were perfused and analyzed for tdTomato expression. **b** Diagram of AAV construct. **c** Diagram showing the injection of AAV into ACC of Drd2-Cre rat. The dashed line of the sagittal section diagram indicates the position of the coronal section. The rectangle indicates the brain regions of ACC. **d** Expression of tdTomato in the ACC from the heterozygous Drd2-Cre rats injected with AAV. Scale bar, 200 μm. Three independent experiments were repeated

to get similar results. **e-f** Immunofluorescent images of tdTomato, GAD67, and NeuN from the rectangles in panel d. Arrowheads indicate Drd2^{+} but Gad67-negative cells, arrows indicate Gad67^{+} but Drd2-negative cells. Scale bar, 25 μm in panel e. Scale bar, 50 μm in panel f. Three independent experiments were repeated to get similar results.

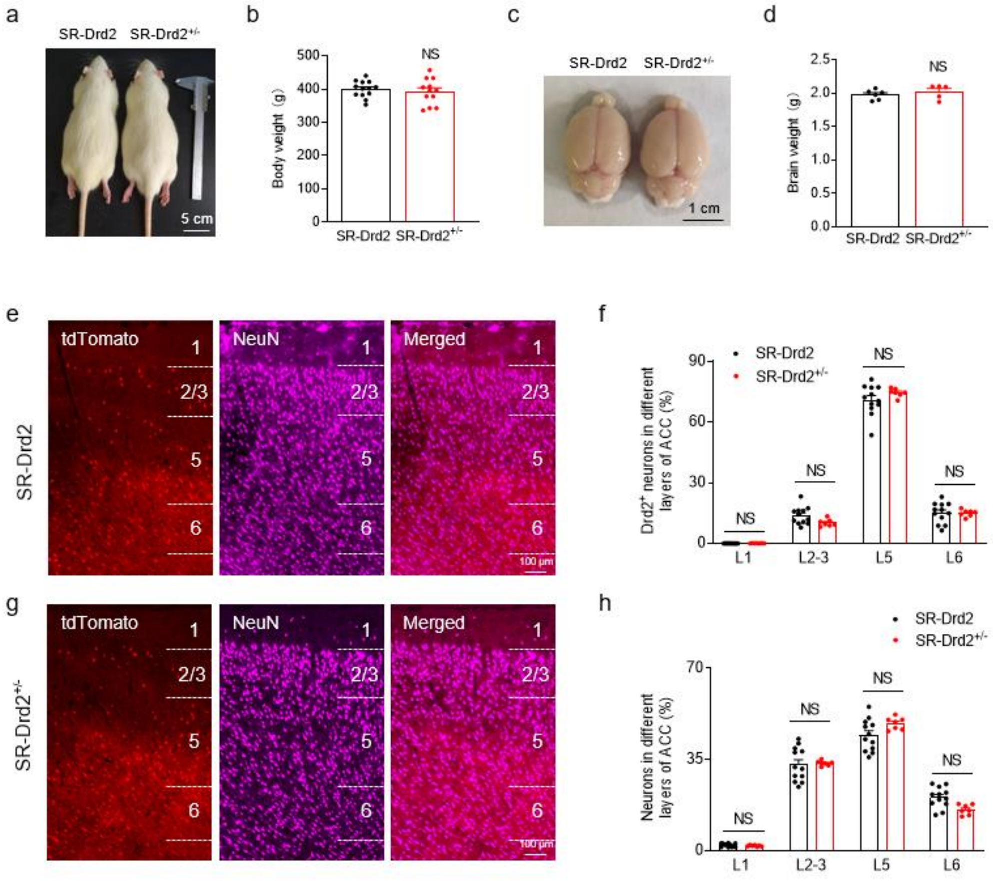

Supplementary Fig. 2 Generation and characterization of SR-Drd2$^{+/-}$ rats.
a Representative image of 8-week-old SR-Drd2 and SR-Drd2$^{+/-}$ rats. Scale bar, 5 cm. **b** Similar body weight between the two groups. NS, not significant, P = 05521, two-sided t test, n = 13 for SR-Drd2, n = 12 for SR-Drd2$^{+/-}$. Data are presented as mean values +/− SEM. **c** Representative brain of 8-week-old SR-Drd2 and SR-Drd2$^{+/-}$ rats. Scale bar, 1 cm. **d** Similar brain weight between the two groups. NS, not significant, P = 0.4237, two-sided t test, n = 6 for SR-Drd2, n = 5 for SR-Drd2$^{+/-}$. Data are presented as mean values +/− SEM. **e** Expression of tdTomato in the ACC from 8-week-old SR-Drd2 rats. Scale bar, 100 μm. **f** The percentage of Drd2^{+} cells in different layers of ACC were similar between the two groups. NS, not significant, two-way ANOVA followed by Sidak's multiple comparisons test, n = 12 for SR-Drd2, n = 6 for SR-Drd2$^{+/-}$. Data are presented as mean values + /− SEM. **g** Expression of tdTomato in the ACC from 8-week-old SR-Drd2$^{+/-}$rats. Scale bar, 100 μm. **h** The percentage of

NeuN$^+$ cells in different layers of ACC were similar between the two groups. NS, not significant, two-way ANOVA followed by Sidak's multiple comparisons test, n = 12 for SR-Drd2, n = 6 for SR-Drd2$^{+/-}$. Data are presented as mean values + /− SEM.

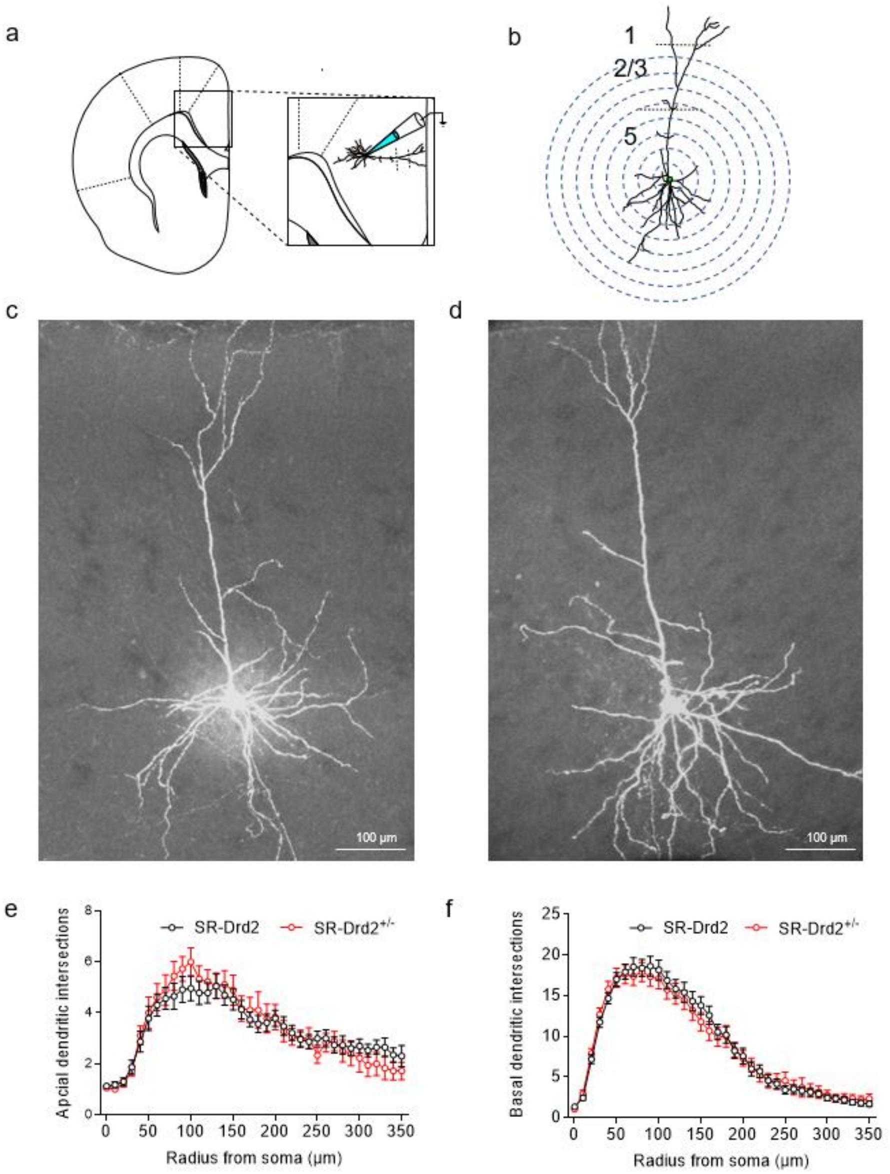

Supplementary Fig. 3 Similar dendritic arborization of Drd2-positive neurons in layer 5 of ACC between SR-Drd2 and SR-Drd2$^{+/-}$ rats.
a Schematic diagram showing the dendrites of Drd2-positive neurons filled with biocytin in layer 5 of ACC. We analyzed the apical and basal dendritic arborization of Drd2-positive neurons. **b** Schematic diagram of Sholl analysis. **c,d** Representative image of Drd2-positive neurons in layer 5 of ACC from SR-Drd2$^{+/-}$ and SR-Drd2 rats. Scale bars, 100 μm. **e** Similar apical dendritic arborization of Drd2-positive neurons in layer 5 of ACC between the two groups. Genotype F (1, 1404) = 0.1139, P = 0.7358, two-way ANOVA, n = 23 neurons from 5 SR-Drd2 rats, n = 18 neurons from

4 SR-Drd2$^{+/-}$ rats. Data are presented as mean values +/− SEM. **f** Similar basal dendritic arborization of Drd2-positive neurons in layer 5 of ACC between the two groups. Genotype F (1, 1169) = 0.3435, P = 0.5579, two-way ANOVA, n = 23 neurons from 5 SR-Drd2 rats, n = 18 neurons from 4 SR-Drd2$^{+/-}$ rats. Data are presented as mean values +/− SEM.

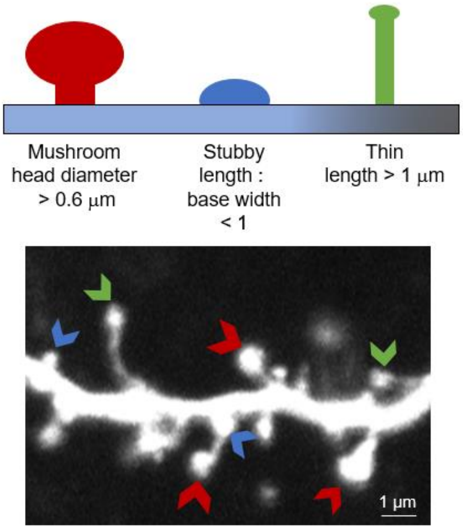

Supplementary Fig. 4 Representative images of different types of spines: mushroom-like (red arrows), stubby (purple arrows), and thin spines (green arrows). Scale bar, 1 µm. Four independent experiments were repeated to get similar results.

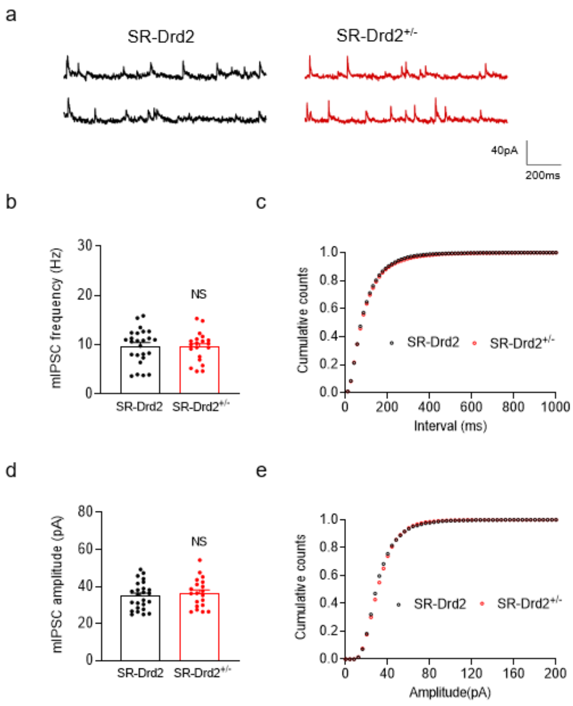

Supplementary Fig. 5 Normal mIPSC of Drd2-positive neurons in layer 5 of ACC from SR-Drd2$^{+/-}$ rats.

a Representative mIPSC traces of Drd2-positive neurons from 8-week-old SR-Drd2 and SR-Drd2$^{+/-}$ rats. **b** Similar mIPSC frequency of Drd2-positive neurons between the two groups. NS, not significant, P = 0.8627, two-sided t test, n = 25 neurons from 5 SR-Drd2 rats, n = 20 neurons from 4 SR-Drd2$^{+/-}$ rats. Data are presented as mean values +/− SEM. **c** Cumulative plots of mIPSC frequency. **d** Similar mIPSC amplitude of Drd2-positive neurons between the two groups. NS not significant, P = 0.5188, two-sided t test, n = 25 neurons from 4 SR-Drd2 rats, n = 20 neurons from 3 SR-Drd2$^{+/-}$ rats. Data are presented as mean values +/− SEM. **e** Cumulative plots of mIPSC amplitude.

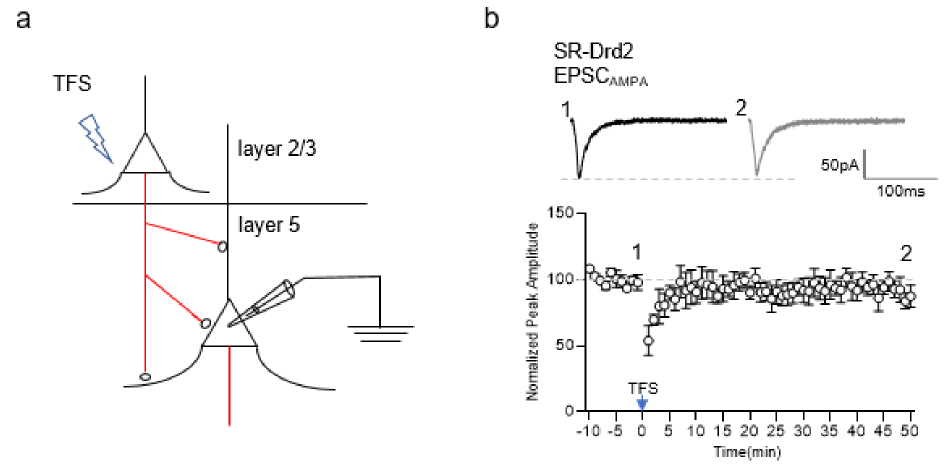

Supplementary Fig. 6 TFS could not induce LTD of AMPAR transmission in layer 5 pyramidal neurons of ACC.

a Schematic diagram showing LTD recordings in layer 5 pyramidal neurons of ACC by application of theta frequency stimulation (TFS) in layers 2-3 of ACC. Red lines indicate axons. The rats used were 4 to 5-week-old age. **b** No LTD of AMPAR-mediated transmission was produced after TFS. Top, representative traces of AMPAR currents before TFS (1) and 50 min after TFS (2). Bottom, normalized peak amplitudes of AMPAR currents from pyramidal neurons of layer 5 ACC were plotted every 1 min in 4 to 5-week-old SR-Drd2 rats. n = 4 neurons from 2 SR-Drd2 rats. Data are presented as mean values +/− SEM.

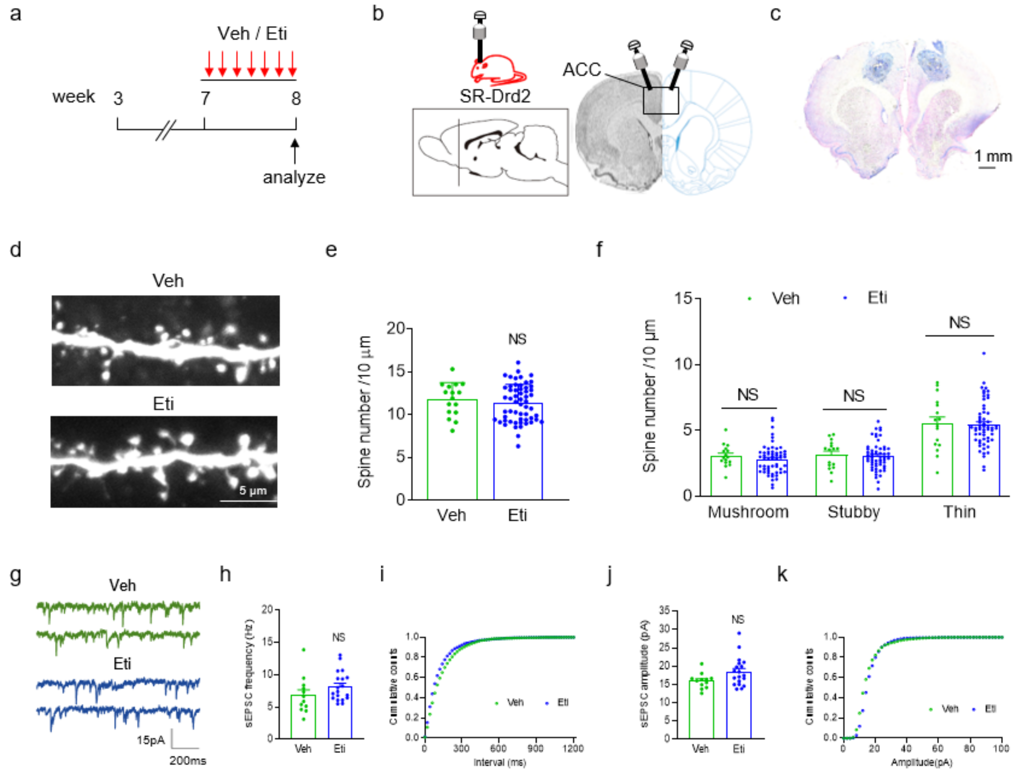

Supplementary Fig. 7 Normal dendritic spine and sEPSC of Drd2-positive neurons after DRD2 inhibition during adulthood.

a Experimental design. SR-Drd2 rats received daily injection of Eti (1 μg in 0.5 μl per side) or Veh (0.5 μl saline per side) into layer 5 of ACC between 7 and 8-week-old age, the dendritic spines and sEPSC in Drd2-positive neurons were then analyzed at 8-week-old age. **b** Diagram showing the injection of Veh or Eti into layer 5 of ACC in SR-Drd2 rat. The dashed line of the sagittal section diagram indicates the position of the coronal section. The rectangle indicates the brain regions of ACC. **c** Nissl staining to determine the injection sites. Scale bar, 1 mm. **d** Representative dendritic spines of Drd2-positive neurons from SR-Drd2 rats treated with Veh or Eti. Scale bar, 5 μm. **e** Normal dendritic spine densities in Drd2-positive neurons after inhibition of Drd2 between 7 and 8-week old age. NS, not significant, $P = 0.4622$, two-sided t test, $n = 16$ dendrites from 3 control rats, $n = 57$ dendrites from 7 Eti-treated rats. Data are presented as mean values +/− SEM. **f** Normal densities of different types of dendritic spines in Drd2-positive neurons after inhibition of Drd2 between 7 and 8-week old age. NS, not significant, P (mushroom) = 0.5555, P (stubby) = 0.6821, P (thin) = 0.6027, two-way-ANOVA followed by Sidak's multiple comparisons test, $n = 16$ dendrites from 3 control rats, $n = 57$ dendrites from 7 Eti-treated rats. Data are presented as mean values +/− SEM. **g** Representative sEPSC traces of Drd2-positive neurons from Veh and Eti-treated SR-Drd2 rats. **h** Similar sEPSC frequency of Drd2-positive neurons between the two groups. NS not significant, $P = 0.1624$, two-sided t test, $n = 12$ neurons from 3 control rats, $n = 19$ neurons from 4 Eti-treated rats. Data

are presented as mean values +/− SEM. **i** Cumulative plots of sEPSC frequency. **j** Similar sEPSC amplitude of Drd2-positive neurons between the two groups. NS, not significant, P = 0.0558, two-sided t test, n =12 neurons from 3 control rats, n = 19 neurons from 4 Eti -treated rats. Data are presented as mean values +/− SEM. **k** Cumulative plots of sEPSC amplitude.

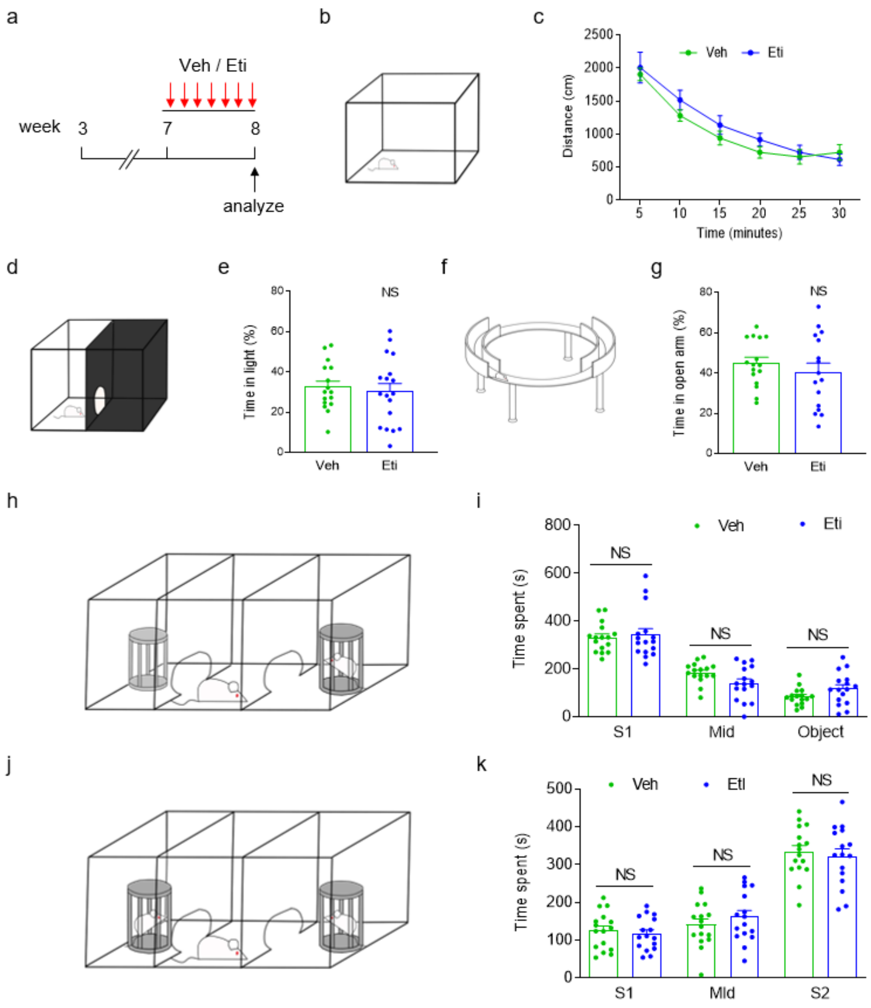

Supplementary Fig. 8 Normal behavior after inhibition of DRD2 in layer 5 of ACC during adulthood

a Experimental design. WT rats received daily injection of Eti (1 μg in 0.5 μl per side) or Veh (0.5 μl saline per side) into layer 5 of ACC between 7 and 8-week-old age, and behaviors were analyzed at 8-week-old age. **b** Schematic diagram of open field. **c** Travel distance in open field was no difference between Eti and Veh-treated rats. NS, not significant, Genotype F (1,258) = 2.527, P = 0.1131, two-way ANOVA, n = 22 for controls, n = 23 for Eti-treated rats. Data are presented as mean values+/− SEM. **d** Schematic diagram of light-dark box. **e** Time spent in light box was similar between the two groups. NS, not significant, P = 0.6683, two-sided t test, n = 16 for controls, n = 18 for Eti-treated rats. Data are presented as mean values +/− SEM. **f** Schematic diagram of elevated maze. **g** Time spent in open arm was similar between the two

groups. NS, not significant, P = 0.4048, two-sided t test, n = 16 for controls, n = 16 for Eti-treated rats. Data are presented as mean values +/− SEM. **h** Schematic diagram of three-chamber test to study social interaction. **i** Social interaction was normal in Eti-treated rats. Time spent in each chamber was quantified. NS, not significant, P (S1) = 0.9547, P (Mid) = 0.1935, P (Object) = 0.4336, two-way ANOVA followed by Sidak's multiple comparisons test, n = 16 for controls, n = 16 for Eti-treated rats. Data are presented as mean values +/− SEM. **j** Schematic diagram of three-chamber test to study social novelty. **k** Social novelty was intact in Eti-treated rats. Time spent in each chamber was quantified. NS, not significant, P (S1) = 0.974, P (Mid) = 0.7322, P (S2) = 0.9287, two-way ANOVA followed by Sidak's multiple comparisons test, n = 16 for controls, n = 16 for Eti-treated rats. Data are presented as mean values +/− SEM.

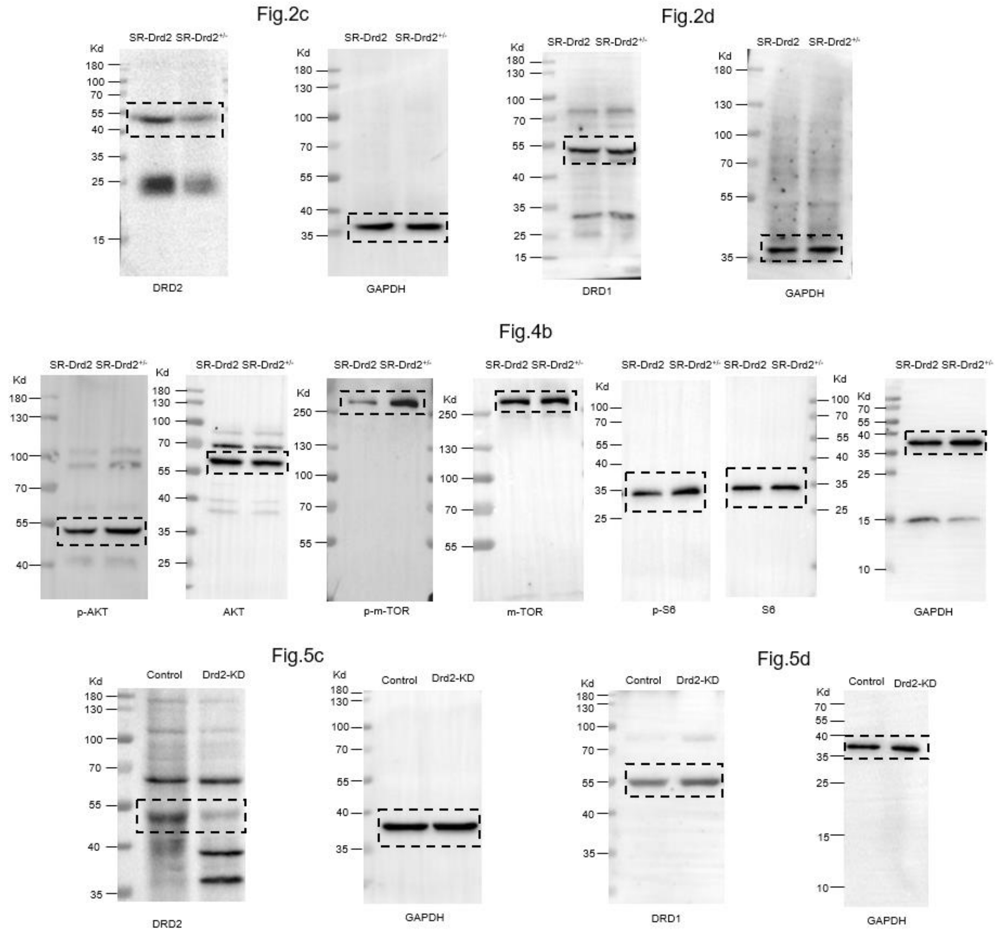

Supplementary Fig. 9 Full scans of representative western blots in all figures. Samples derive from the same experiment and blots were processed in parallel.
