# Supplementary material for: Dopamine D2 receptor regulates cortical synaptic pruning in rodents
Source: Nat Commun. 2021 Nov 8;12:6444. doi: 10.1038/s41467-021-26769-9 (PMC8576001; doi:10.1038/s41467-021-26769-9)
Supplement: Supplementary file 3 — Reporting Summary [file 41467_2021_26769_MOESM3_ESM.pdf]

nature research

Corresponding author(s): D Y

Last updated by author(s): Sep 11, 2021

Reporting Summary

Nature Research wishes to improve the reproducibility of the work that we publish. This form provides structure for consistency and transparency in reporting. For further information on Nature Research policies, see our Editorial Policies and the Editorial Policy Checklist.

Statistics

For all statistical analyses, confirm that the following items are present in the figure legend, table legend, main text, or Methods section.

n/a	Confirmed	
☐	☒	The exact sample size (n) for each experimental group/condition, given as a discrete number and unit of measurement
☐	☒	A statement on whether measurements were taken from distinct samples or whether the same sample was measured repeatedly
☐	☒	The statistical test(s) used AND whether they are one- or two-sided<br>*Only common tests should be described solely by name; describe more complex techniques in the Methods section.*
☐	☒	A description of all covariates tested
☐	☒	A description of any assumptions or corrections, such as tests of normality and adjustment for multiple comparisons
☐	☒	A full description of the statistical parameters including central tendency (e.g. means) or other basic estimates (e.g. regression coefficient) AND variation (e.g. standard deviation) or associated estimates of uncertainty (e.g. confidence intervals)
☐	☒	For null hypothesis testing, the test statistic (e.g. F, t, r) with confidence intervals, effect sizes, degrees of freedom and P value noted<br>*Give P values as exact values whenever suitable.*
☒	☐	For Bayesian analysis, information on the choice of priors and Markov chain Monte Carlo settings
☒	☐	For hierarchical and complex designs, identification of the appropriate level for tests and full reporting of outcomes
☒	☐	Estimates of effect sizes (e.g. Cohen's d, Pearson's r), indicating how they were calculated

Our web collection on statistics for biologists contains articles on many of the points above.

Software and code

Policy information about availability of computer code

Data collection	TissueFAX Plus ST (Tissue Gnostics), ChemiDocTM XRS + Imaging System (Bio-Rad), Leica TCS SP8 confocal microscope, Plexon MAP system, Vibroslice (VT 1000S; Leica), Olympus BX51WI microscope, HEKA EPC 10 double patch-clamp amplifier, PATCHMASTER version 2 x 90.5 software (HEKA), Stereotaxic apparatus (RWD Life Science), Stereotaxic injector (Stoelting).
Data analysis	Image J (NIH), Simple Neurite Tracer (v1.53c), Reconstruct software (v1.1.0.0), Mini Analysis Program (Synaptosoft), Igor pro 6.7.3.2 (WaveMetrics) with Neuromatic version 3.0, True-Scan System (Coulbourn Instruments), ANY-maze video tracking system (Stoelting), GraphPad Prism 8 (GraphPad).

For manuscripts utilizing custom algorithms or software that are central to the research but not yet described in published literature, software must be made available to editors and reviewers. We strongly encourage code deposition in a community repository (e.g. GitHub). See the Nature Research guidelines for submitting code & software for further information.

Data

Policy information about availability of data

All manuscripts must include a data availability statement. This statement should provide the following information, where applicable:

- Accession codes, unique identifiers, or web links for publicly available datasets
- A list of figures that have associated raw data
- A description of any restrictions on data availability

All data supporting the results presented herein are available from the article paper, supplementary information and source data. The full-length images for all the gels and blots are provided in Supplementary Fig. 9. Source data are provided with this paper.

Field-specific reporting

Please select the one below that is the best fit for your research. If you are not sure, read the appropriate sections before making your selection.

- [x] Life sciences
- [ ] Behavioural & social sciences
- [ ] Ecological, evolutionary & environmental sciences

For a reference copy of the document with all sections, see nature.com/documents/nr-reporting-summary-flat.pdf

Life sciences study design

All studies must disclose on these points even when the disclosure is negative.

Sample size	Sample size choice was based on previous studies (Wang et al., Nature Communications, 2021, doi: 10.1038/s41467-020-20552-y), not predetermined by a statistical method.
Data exclusions	The outliers were calculated with the Grubbs's test. One data point in the experiments of elevated O maze (Fig. 9g) was excluded per this criteria.
Replication	Independent experiments were repeated three to four times to get the similar results.
Randomization	Randomized, some animals where not randomized due to the necessity of a genetic construct (SR-Drd2, SR-Drd2+/- rats).
Blinding	The investigators were blind to the genotype and treatment of the animals.

Reporting for specific materials, systems and methods

We require information from authors about some types of materials, experimental systems and methods used in many studies. Here, indicate whether each material, system or method listed is relevant to your study. If you are not sure if a list item applies to your research, read the appropriate section before selecting a response.

Materials & experimental systems

n/a	Involved in the study	
[ ]	[x]	Antibodies
[x]	[ ]	Eukaryotic cell lines
[x]	[ ]	Palaeontology and archaeology
[ ]	[x]	Animals and other organisms
[x]	[ ]	Human research participants
[x]	[ ]	Clinical data
[x]	[ ]	Dual use research of concern

Methods

n/a	Involved in the study	
[x]	[ ]	ChIP-seq
[x]	[ ]	Flow cytometry
[x]	[ ]	MRI-based neuroimaging

Antibodies

Antibodies used	rabbit anti-NeuN (1:500, Abcam, ab177487), rabbit anti-GAPDH (1:5000, Abways, ab0037), mouse anti-GAD67 (1:300, Millipore, MAB5406, clone 1G10.2), rabbit anti-DRD2 (1:1000, Millipore, AB15588), rat anti-DRD1 (1:200, Sigma, D2944), rabbit anti-p-AKT (1:1000, arigo, ARG51558), rabbit anti-AKT (1:1000, GeneTex, GTX121937), rabbit anti-p-mTOR (1:1000, Cell Signaling Technology, 2974), rabbit anti-mTOR (1:1000, Cell Signaling Technology, 2983), rabbit anti-p-S6 ribosomal protein (Ser235/236) (1:1000, Cell Signaling Technology, 2211), rabbit anti-S6 ribosomal protein (1:1000, Cell Signaling Technology, 2217), Alexa Fluor-488 goat-anti-mouse secondary antibodies (A11029; 1:500, Thermo Fisher), Alexa Fluor-647 goat-anti-rabbit secondary antibodies (A21244; 1:500, Thermo Fisher), HRP-conjugated secondary antibody (goat-anti-rabbit, G-21234, 1:2000, Thermo Fisher), rabbit-anti-rat IgG (1:500, Sangon, D111017), HRP-conjugated secondary antibody (goat-anti-rabbit, G-21234, 1:2000, Thermo Fisher), Alexa Fluor-488 conjugated streptavidin (S32354; 1:200, Thermo Fisher).
Validation	The specificity of the following antibodies was validated for immunostaining in rats by the manufacturers: anti-NeuN and anti-GAD67. The specificity of the following antibodies was validated for western blots in rats by the manufacturers: anti-DRD1, anti-DRD2, anti-GAPDH, anti-p-AKT, anti-AKT, anti-p-mTOR, anti-mTOR, anti-p-S6, and anti-S6. All antibodies used can be found on the corresponding websites by catalog number.

Animals and other organisms

Policy information about studies involving animals; ARRIVE guidelines recommended for reporting animal research

Laboratory animals	We used WT, Drd2-Cre, Ai14, Drd2 heterozagous rats in this study. All rats are in Sprague-Dawley (SD) backgroud. They are male rats aged at 2 weeks, 3 weeks, 4 weeks, 5 weeks, 7 weeks and 8 weeks. Animals were housed in rooms at 23°C and 50% humidity in a 12 hr light/dark cycle and with food and water available ad libitum.

Wild animals	No wild animals were used in the study.
Field-collected samples	No field collected samples were used in the study.
Ethics oversight	All experimental procedures were reviewed and approved by the Institutional Animal Care and Use Committee of East China Normal University.

Note that full information on the approval of the study protocol must also be provided in the manuscript.
